# The association between obesity and dengue severity among pediatric patients: A systematic review and meta-analysis

**DOI:** 10.1371/journal.pntd.0006263

**Published:** 2018-02-07

**Authors:** Mohd Syis Zulkipli, Maznah Dahlui, Nor’ashikin Jamil, Devi Peramalah, Hoe Victor Chee Wai, Awang Bulgiba, Sanjay Rampal

**Affiliations:** 1 Julius Centre University of Malaya, Department of Social and Preventive Medicine, Faculty of Medicine, University of Malaya, Kuala Lumpur, Malaysia; 2 Ministry of Health Malaysia, Putrajaya, Malaysia; University of Heidelberg, GERMANY

## Abstract

**Background:**

Severe dengue infection often has unpredictable clinical progressions and outcomes. Obesity may play a role in the deterioration of dengue infection due to stronger body immune responses. Several studies found that obese dengue patients have a more severe presentation with a poorer prognosis. However, the association was inconclusive due to the variation in the results of earlier studies. Therefore, we conducted a systematic review and meta-analysis to explore the relationship between obesity and dengue severity.

**Methods:**

We performed a systematic search of relevant studies on Ovid (MEDLINE), EMBASE, the Cochrane Library, Web of Science, Scopus and grey literature databases. At least two authors independently conducted the literature search, selecting eligible studies, and extracting data. Meta-analysis using random-effects model was conducted to compute the pooled odds ratio with 95% confidence intervals (CI).

**Findings:**

We obtained a total of 13,333 articles from the searches. For the final analysis, we included a total of fifteen studies among pediatric patients. Three cohort studies, two case-control studies, and one cross-sectional study found an association between obesity and dengue severity. In contrast, six cohort studies and three case-control studies found no significant relationship between obesity and dengue severity. Our meta-analysis revealed that there was 38 percent higher odds (Odds Ratio = 1.38; 95% CI:1.10, 1.73) of developing severe dengue infection among obese children compared to non-obese children. We found no heterogeneity found between studies. The differences in obesity classification, study quality, and study design do not modify the association between obesity and dengue severity.

**Conclusion:**

This review found that obesity is a risk factor for dengue severity among children. The result highlights and improves our understanding that obesity might influence the severity of dengue infection.

## Author summary

Dengue fever, an Aedes mosquito-borne viral infection, is a self-limiting or asymptomatic viral infection. In its severe form, severe dengue, the symptoms include organ failure, life-threatening bleeding, and shock. Patients with obesity were found to be at higher risks of developing complications and severe dengue infection compared to non-obese patients. Our systematic review and meta-analysis critically compared the available shreds of evidence related to obesity and severe forms of dengue infection. A total of fifteen studies that include nine cohort studies, five case-control studies, and one cross-sectional study were reviewed to explore the association between obesity and dengue severity. The results indicated that obese patients have higher odds of developing severe dengue infection compared to non-obese patients. This review highlighted the importance of obesity in the progression of dengue infection. With increasing prevalence of obese individual as well as expanding areas that were at high risk for dengue infection, obesity may play a role in increasing the burden and mortality related to dengue infection. Further large-scale prospective studies in regions with high prevalence of dengue infections would provide additional information and some better discernment on the relevance of obesity in dengue infection.

## Introduction

Dengue is an acute systemic viral infection caused by the dengue virus. Currently, there are four known dengue virus subtypes (DEN-1, DEN-2, DEN-3, DEN-4) with *Aedes aegypti* and *Aedes albopictus* as the principal vectors. The burdens of dengue infection were concentrated in the tropical and sub-tropical regions mainly in Asia, Caribbean, Africa, the Pacific, and the Americas.

Globally, an annual average of 9,221 fatal dengue cases was recorded between 1990 and 2013 [1]. In 2013 alone, dengue infection was responsible for 576,900 years-life-lost (YLL) and 1.14 million disability-adjusted-life-years (DALY) [1]. Only 25% of those infected with dengue were symptomatic [2]. The signs and symptoms of dengue infection include fever, nausea, vomiting, joint pain, muscular pain, retro-orbital pain, and spontaneous bleeding. The most important predictor of adverse dengue infection was the rapid decrease of platelets with increased hematocrit levels during the critical phase.

Currently, the most commonly used dengue classification is the WHO 2009 dengue classification. The WHO 2009 classification classified dengue into Dengue without Warning Signs (DWWS), Dengue with Warning Signs (DWS), and Severe Dengue (SD) [3]. Previously, dengue infection was classified using the WHO 1997 classification. The WHO 1997 classified dengue differently from WHO 2009 into Dengue Fever (DF), Dengue Hemorrhagic Fever (DHF), and Dengue Shock Syndrome (DSS). Studies have found that the WHO 2009 dengue classification into severity levels was 53 percent more sensitive in capturing severe dengue infection than the WHO 1997 dengue classification [4, 5]. Other dengue classifications include WHO 1986, 1999, and 2011.

Epidemiological studies have recognized several risk factors for severe dengue infection. Among the risk factors were the two extremes of age, under-nutrition, pregnancy, and secondary infections [6–10]. Furthermore, there were specific clinical signs and symptoms of acute dengue infection that links to severe dengue such as abdominal pain, vomiting, spontaneous bleeding, and diarrhea [11–13]. Nonetheless, other potential risk factors may have a role as novel predictors for dengue severity such as obesity, diabetes, chronic allergies, and hypertension [10, 14–18].

Overweight and obesity defined as abnormal or excessive fat accumulation that presents a risk to health. Depending on age, different methods were available to measure a body’s healthy weight according to age groups. We defined overweight and obesity using the WHO standards. In children and adolescents using age and sex cut-offs: (1) overweight defined as either body mass index (BMI) ≥ 85^th^ percentile and < 95^th^ percentile or weight-for-height percentage of > 110%; (2) obesity defined as either BMI ≥ 95th percentile or weight-for-height percentage of more than 120%. In adults, overweight defined as BMI ≥ 25 kg/m^2^ and < 30 kg/m^2^, and obesity was defined as ≥ 30 kg/m^2^ [19].

Obesity is a major Public Health problem. Globally, the prevalence of overweight and obesity rose by 27.5 percent for adults between 1980 and 2013. The number increased from 857 million in 1980 to 2.1 billion in 2013 [20]. In 2016, the WHO reported that the prevalence of overweight and obesity were at: (i) 21.9% and 4.7% in South-East Asia region; (ii) 49% and 20.8% in Eastern Mediterranean region; (iii) 31.7% and 6.4% in Western Pacific region; (iv) 31.1% and 10.6% in African region; and (v) 62.5% and 28.6% in Americas region, respectively [21, 22]. An estimate by Brady et al. on the prevalence of dengue showed that 3.9 billion people in 128 countries are at risk for dengue virus infection [23]. WHO also reported that dengue has spread to new areas such as France, Europe, China, and Japan [24].

Hypothetically, obesity may affect the severity of dengue infection through the inflammation pathways. The increased deposition of white adipose tissue (WAT) in the overweight and obese individual leads to increase production of inflammatory mediators that were known to increase the capillary permeability and causes plasma leakage [25–27].

In 2013, Huy NT et al. published a systematic review and meta-analysis of 198 studies up to September 2010 on factors associated with dengue shock syndrome. In the sub-analysis of eight primary studies, the author found that obesity was no association between DSS and overweight or obesity [28]. Recently, in 2016, Trang et al. published a systematic review and meta-analysis of thirteen studies up to August 2013 on the association between nutritional status and dengue infection [29]. The meta-analysis of eight primary studies on overweight and obesity found no significant association between overweight/obese and DSS. However, the author failed to show substantial consistency regarding the relationship between studies and concluded that the effects of nutritional status on dengue outcomes were controversial. Similarly, both systematic reviews by Huy NT et al. and Trang et al. focused only on the association between nutritional status and dengue infection with studies up to August 2013.

There were scarce and inconclusive shreds of evidence linking overweight and obesity with the severity of dengue infection [30, 31]. The number obese individuals susceptible to dengue will significantly increase with the increasing prevalence of obesity and increasing populations susceptible to dengue infection. With the hypothesized link between obesity and increasing plasma leakage, obese individuals may be at higher risk of developing severe dengue infection. Our review aims to summarize the current evidence on the association between obesity and severe dengue infection and to identify patients with high risk of severe infection. To our knowledge, this is the first systematic review and meta-analysis on the association between obesity and dengue severity.

## Materials and methods

We conducted this systematic review and meta-analysis according to the recommendations of the Preferred Reporting Items for Systematic Reviews and Meta-Analyses (PRISMA) statement [32]. We had registered the protocol for this study with the PROSPERO International prospective register of systematic reviews (No. CRD: 42016046944).

### Literature searching and selection criteria

#### Review question and search strategy

Using the P.I.C.O strategy (an acronym for population or problem, intervention or exposure of interest, comparison, and outcome) for framing review question [33], we framed the review question as: "do the severity of dengue infection associated with obesity?" Based on the review question, we developed a search strategy for each database using the search terms as described in Table 1. In total, we searched five databases, i.e., Ovid (MEDLINE), the Cochrane Library, EMBASE, Web of Science, and Scopus for relevant studies.

**Table 1:** Search strategy including search terms and databases.

Database	Search Terms
**Ovid (Medline)**	dengue.sh., dengue.ti., dengue.ab., adipos*.sh., adipos*.ti., adipos*.ab., obes*.sh., obes*.ti., obes*.ab., body mass index/.sh., body mass index/.ti., body mass index/.ab., bmi.sh., bmi.ti., bmi.ab., waist circumference*/.sh., waist circumference*/.ti., waist circumference*/.ab., nutriti*.sh., nutriti*.ti., nutriti*.ab., malnutriti*.sh., malnutriti*.ti., malnutriti*.ab., flavi*.sh., flavi*.ti., flavi*.ab., #1 or #2 or #3, #4 - #27/or, #28 and #29, limit 30 to humans.
**Embase**	dengue, 'dengue'/exp, adipos*, 'adiposity'/exp, obes*, 'obesity'/exp, 'body mass index', 'body mass index'/exp, bmi, 'waist circumference*', 'waist circumference'/exp, nutriti*, 'nutrition'/exp, malnutriti*, 'malnutrition'/exp, flavi*, 'flavivirus'/exp, #1 OR #2, #3 - #17/OR, #18 AND #19, #20 AND [humans]/lim.
**Cochrane**	dengue:ti,ab,kw, adipos*:ti,ab,kw, obes*:ti,ab,kw, 'body mass index':ti,ab,kw, bmi:ti,ab,kw, 'waist circumference*':ti,ab,kw, nutriti*:ti,ab,kw, malnutriti*:ti,ab,kw, flavi*:ti,ab,kw, #2 - #9/or, #1 and #10.
**Scopus**	(TITLE-ABS-KEY(dengue)), (TITLE-ABS-KEY(adipos*)), (TITLE-ABS-KEY(obes*)), (TITLE-ABS-KEY ('body mass index')), (TITLE-ABS-KEY(bmi)), (TITLE-ABS-KEY ('waist circumference')), (TITLE-ABS-KEY(nutriti*)), (TITLE-ABS-KEY(malnutriti*)), (TITLE-ABS-KEY(flavi*)), #2 - #9/OR, #1 AND #10, #11 AND (LIMIT-TO (EXACTKEYWORD, "Human")).
**Web of Science**	TS = dengue, TI = dengue, TS = adipos*, TI = adipos*, TS = obes*, TI = obes*, TS = 'body mass index', TI = 'body mass index', TS = bmi, TI = bmi, TS = 'waist circumference', TI = 'waist circumference', TS = nutriti*, TI = nutriti*, TS = malnutriti*, TI = malnutriti*, TS = flavi*, TI = flavi*, #1 OR #2, #3 - #18/OR, #19 AND #20, #19 AND #20 NOT TS = animal.

We searched four databases with a date of search from the inceptions until July 12, 2017. Due to administrative issues, we also managed to search EMBASE from their inception until September 21, 2016. Furthermore, we explored the keyword "dengue" on other remaining libraries including the New York Academy of Medicine Grey Literature Report (NYAM), African Index Medicus (AIM), System for Information on Grey Literature in Europe (SIGLE) and African Journals OnLine (AJOL). We also screened the references of relevant reviews manually to find related articles for our review [28, 29].

### Inclusion and exclusion criteria

We included all interventional and observational studies that evaluated the association between obesity and dengue outcomes. We also added any studies that have information on dengue patients together with information on body compositions such as weight, height, body mass index (BMI) and waist circumference (WC). We excluded articles with the following characteristics: (i) letter, case report, review, or conference paper; (ii) animal study; and (iii) in vitro study without patients. MSZ and NAJ independently examined and screened the titles and abstracts for eligibility. There is no restriction with regards to age and publication language.

### Screening

We imported the relevant articles retrieved from the searches to EndNote X7.7.1 software (Thomson Reuters, Philadelphia). Duplicates were removed primarily using the EndNote software and manually by identification of similar title, author, and the year before the screening process. MSZ also translated one Indonesian article into English during the screening process. Next, the remaining studies were carefully appraised from title and abstract to identify those that may be related to the review question. We based the identification process on the PRISMA flowchart [32]; i.e., identification, screening, eligibility, and study inclusion. We resolved any disagreements in selection by consensus between authors.

### Data extraction

We acquired the full-text version of all eligible studies from its databases digitally. MSZ and NAJ reviewed all available full text before data extraction independently. We developed a Google form for data extraction that includes information on the first author, publication year, recruitment, study design, data collection process, allocation of patients (sequential or random), and study site. The form also included characteristics of the patient population, sample size, dengue criteria), index of obesity (BMI, BMI Z-score, BMI-for-age, weight, weight-for-age, height, waist circumference), measurement tools for body composition, and age distributions of the patients. Data were then extracted independently by MSZ and NAJ, and verified by SR and MD.

The outcome measured in this review was severe dengue infection compared to non-severe dengue infection. We defined severe dengue infection as a collective term that includes either DHF grade III, DHF grade IV, dengue with warning signs, DSS, or severe dengue. Non-severe dengue group includes either DF, DHF grade I, DHF grade II, or dengue without warning signs. The exposure was defined as obesity that includes overweight. The reference group of exposure defined as non-obese includes patients with normal weight and underweight.

### Quality assessment

The quality of the included studies was appraised using the Newcastle-Ottawa Scale (NOS) [34]. The NOS evaluated study quality based on three quality parameters (selection, comparability, and outcome) divided across eight specific items. Each item on the scale was scored from one point to score up to two points. Currently, there was no specific cut-off score for stratification of study into good, poor, and moderate. Based on our literature search, we found that a cut-off score of seven or more was used to consider a study of good quality. A score between five and six was considered as moderate quality study and anything lesser than five was considered as a poor quality study [35]. Thus, we adopted the similar cut-off score and pair the score with traffic light color coding as described in S1 Table for assessment of study quality in this review.

### Statistical analysis

We conducted the meta-analysis using Stata 14 (StataCorp, Texas) and considered p<0.05 as statistically significant. We used random-effects model for meta-analysis of the association between obesity and dengue severity. The random-effect compared to fixed-effect model is a more robust model by accounting for the variation between the actual effect estimates of included studies. Before pooling the odds ratio estimate, we calculated the individual odds ratio of developing severe dengue infection among obese patients compared to non-obese patients using the available information extracted from the included studies. We computed the pooled odds ratio estimate by weighting each estimate using the inverse variance method. Forest plot and I^2^ statistic were used to illustrate the combined estimate and evaluate heterogeneity, respectively.

In our sensitivity analyses, we used meta-regression to explore the effect of heterogeneity by obesity category (overweight/obesity and obesity), study quality (good, moderate, poor), and study design (cohort, case-control, cross-sectional). Publication bias was assessed using a funnel plot and Egger’s Test. We assumed missingness of any outcomes to be missing completely at random.

## Results

### Study selection process

**Fig 1:**
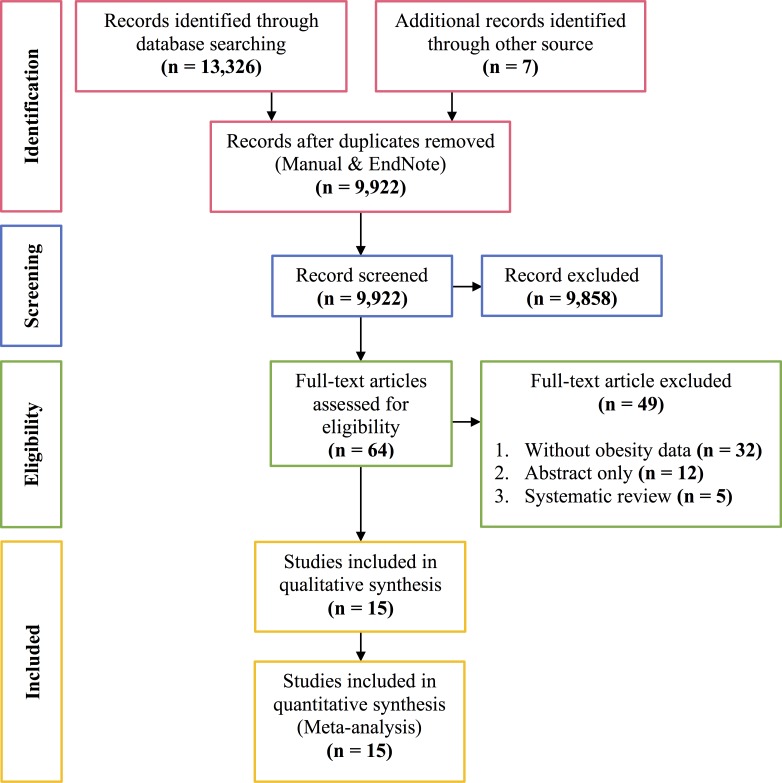
PRISMA 2009 Flow Diagram.

We identified a total of 13,333 potential reports from five databases and references screening of three earlier relevant reviews [28, 29], with 3,411 duplicate reports. We included sixty-four reports for the full-text review. Furthermore, we excluded forty-nine reports that did not fulfill the inclusion and exclusion criteria. Finally, we included fifteen reports for systematic review and meta-analysis. The process of study selection was presented in the PRISMA 2009 Flow Diagram (Fig 1) [32].

### Descriptive analysis

#### Designs, settings, and subjects

The fifteen studies were from 2000 to 2016 [10, 31, 36–48]. Table 2 summarized all available information of the included studies presented in Table 2. Most studies were conducted in Asia (seven from Indonesia, five from Thailand, and one from Sri Lanka) [10, 31, 36–43, 45, 46, 48] while another two articles were from El Salvador and Paraguay [44, 47]. There were nine cohort studies [36–39, 42, 44, 45, 47, 48], five case-control studies [10, 31, 40, 41, 46], and one cross-sectional study [43]. All fifteen studies only included pediatric patients as subjects.

**Table 2:** The summary of included studies.

First Author, Year, Country, Sample Size, Study Design	Main Objectives	Selection Criteria	Methods and Results				
Chuansumrit et al., 2000, Thailand, N = 175 patients, Cohort [36].	To determine the transfusion requirements among patients with dengue infection.	**Inclusion criteria:** Patients diagnosed with dengue infection.	**Methods:** This study uses the WHO 1986 dengue classification. The exposures were determined using the weight-for-age percentile. There were 44 patients in the cases group (dengue hemorrhagic fever III and IV) and 116 patients in the control group (dengue hemorrhagic fever grade I and II). 15 Dengue Fever patients were excluded from the analysis. **Definition of Exposures: a. Overweight =** Weight-for-age > 75 percentiles.				
		**Exclusion criteria:** Not mentioned	**Results:** The mean body weight was at the 54th percentile for age. The patients whose body weight exceeded the 50th percentile for age had a higher chance to develop grade III or IV DHF than those with lesser body weight (p = 0.039).				
			**Exposure**	**Cases (%)**	**Control (%)**		
			Body Weight < 25 Percentile	9 (23.7%)	29 (76.3%)		
			Body Weight > 25–50 Percentile	6 (13.9%)	37 (86%)		
			Body Weight > 50–75 Percentile	12 (44.4%)	15 (55.6%)		
			Body Weight > 75 percentile	17 (32.7%)	35 (67.3%)		
Basuki, 2003, Indonesia, N = 41 patients, Cohort [37].	To identify the level of von Willebrand factor in dengue infection and correlate the increase in vWF with thrombocytopenia.	**Inclusion Criteria:** Suspected dengue infections admitted to the Pediatric Ward Dr. Soetomo Hospital, from Sept '00 to '01.	**Methods:** This study uses the WHO 1997 dengue classification. 41 patients include dengue fever, dengue hemorrhagic fever grade I, II, and III. **Definition of Exposures:** Not mentioned.				
		**Exclusion Criteria:** Not mentioned	**Results:** 56.1% of patients have normal weight, 29.3% malnutrition and 14.6% obese.				
			**Exposure**	**DF (%)**	**DHF I (%)**	**DHF II (%)**	**DHF III (%)**
			Malnutrition	1 (25%)	1 (50%)	3 (60%)	7 (23.3%)
			Normal	3 (75%)	0 (0%)	2 (40%)	18 (60%)
			Obesity	0 (0%)	1 (50%)	0 (0%)	5 (16.7%)
Kan et al., 2004, Indonesia, N = 85 patients, Cohort [38].	To find out factors associated with shock in DHF.	**Inclusion Criteria:** Hospitalized patients who met WHO criteria for DHF with positive ELISA.	**Methods:** This study uses the WHO 1997 dengue classification, and the exposure was determined using weight-for-age. There were 42 patients in the shock group (patient with dengue hemorrhagic fever grade III and IV) and 43 patients in the no shock group (patient with dengue hemorrhagic fever grade I and II). **Definition of Exposures: a. Overweight =** Weight-for-age > 75 percentiles.				
		**Exclusion Criteria:** Any other infectious disease or their parents did not give informed consent.	**Results:** Logistic regression analysis found that duration of fever (p = 0.001), abdominal pain (p = 0.009), hematocrit level (p = 0.005), and platelet count (p = 0.003) constituted independent factors correlating with shock in DHF.				
			**Exposure**	**Shock (%)**	**No Shock (%)**		
			Underweight	15 (36%)	16 (37%)		
			Normal	22 (52%)	21 (49%)		
			Overweight	5 (12%)	6 (14%)		
Malavige et al., 2006, Sri Lanka, N = 104 patients, Cohort [39].	To describe patterns of clinical disease in a cohort of children hospitalized with dengue during the major dengue epidemic in Sri Lanka.	**Inclusion Criteria:** Children with clinical features suggestive of dengue infections admitted to the general pediatric ward at Lady Ridgeway Hospital in Colombo.	**Methods:** This study uses the WHO 1999 dengue classification, and the exposure was determined using BMI-for-age centiles. There were 52 patients in the case group (patient with dengue hemorrhagic fever grade II, III, and IV) and 52 patients in the control group (patient with dengue fever, dengue hemorrhagic fever grade I). **Definition of Exposures: a. Obesity =** BMI-for-age > 90 centiles.				
		**Exclusion Criteria:** Not mentioned	**Results:** Age, sex, the presence of atopic diseases (such as asthma or eczema), and nutritional status did not appear to alter the risk for severe dengue infections.				
			**Exposure**	**Mild (%)**	**Severe (%)**	**OR (95% CI)**	**P**
			BMI-for-age > 90th centile	3 (5.8%)	4 (7.7%)	1.40 (0.30, 6.50)	>0.05
			BMI-for-age < 5^th^ centile	28 (53.8%)	27 (51.9%)	0.90 (0.40, 2.20)	>0.05
Bongsebandhu et al., 2008, Thailand, N = 98 patients, Cohort [42].	To analyze potential risk factors and study their correlation with severity of dengue infection.	**Inclusion Criteria:** 0–15 years of age, suspected dengue infection admitted to King Chulalongkorn Memorial Hospital from Oct '04 to Sept '06.	**Methods:** This study uses the WHO 1997 dengue classification, and the exposure was determined using the weight-for-age percentage of ideal body weight (IBW). There were 52 patients in the severe infection group (patient with dengue hemorrhagic fever) and 46 patients in the non-severe group (patient with dengue fever). **Definition of Exposures: a. Obese =** >110% ideal body weight-for-age standard of Thai children.				
		**Exclusion Criteria:** Patients with prior coagulation disorders or those proven not infected with dengue virus.	**Results:** Ninety-four patients (95.9%) were of normal nutritional status or obese (body weight 75–110% or >110% of the ideal body weight of Thai patients in the same age. It was inconclusive as to whether nutritional status was a risk factor.				
			**Exposure**	**Non-severe (%)**	**Severe (%)**		
			Undernourished	2 (4.3%)	2 (3.8%)		
			Normal	21 (45.7%)	22 (42.3%)		
			Obese	23 (50%)	28 (53.8%)		
Maron et al., 2010, El Salvador, N = 202 patients, Cohort [44].	To examine the nutritional status of children in El Salvador and its relationship between this and the severity of dengue infection.	**Inclusion Criteria:** Children age 5–12 years, with DHF/DF, prospectively identified from Infectious Diseases Ward, Intensive Care Unit, and Dengue Service Area.	**Methods:** This study uses the WHO 1997 dengue classification, and the exposures were determined using the weight-for-age and BMI-for-age Z-score. There were 62 patients in the DHF group (patient with dengue hemorrhagic fever), 66 patients in the DF (patient with dengue fever), and 74 patients in the control group (healthy control matched for neighborhoods of residence from schools attended by cases). **Definition of Exposures: a. Overweight =** BMI-for-age Z-score (BAZ) > + 2 SD.				
		**Exclusion Criteria:** Patients with known immunodeficiency.	**Results:** There were no differences in weight-for-age Z-score (WAZ) or BMI-for-age Z-scores (BAZ) between the three groups. Children with dengue fever had a greater height-for-age than healthy controls but no significant differences in rates of stunting. There was no difference in height between children with dengue fever and dengue hemorrhagic fever. Excess nutrition does not appear to be a risk factor for severe forms of dengue infection in El Salvador, nor does malnutrition seem to be predictive of good outcomes.				
			**Exposure**	**DHF (%)**	**DF (%)**		
			Normal	38 (86.3%)	48 (92.3%)		
			Malnourished (WAZ < -2 SD)	3 (6.8%)	0 (0.0%)		
			Overweight (WAZ > +2 SD)	3 (6.8%)	4 (7.7%)		
			Normal	45 (72.6%)	56 (84.9%)		
			Malnourished (BAZ < -2 SD)	5 (8.1%)	1 (1.5%)		
			Overweight (BAZ > +2 SD)	12 (19.4%)	9 (13.6%)		
Widiyati et al., 2013, Indonesia, N = 342 patients, Cohort [45].	To evaluate childhood obesity as a prognostic factor for DSS.	**Inclusion Criteria:** Age 18 years and below, fulfilled criteria for DHF/DSS, admitted to Sardjito Hospital from June '08 to Feb '11.	**Methods:** This study uses the WHO 1997 dengue classification and the exposure was determined using the BMI-for-age Z-score (BAZ). There were 116 patients in the case group (patient with dengue hemorrhagic fever grade III and IV) and 226 patients in the control group (patient with dengue hemorrhagic fever grade I and II). **Definition of Exposures: a. Obese =** BMI-for-age Z-score (BAZ) > + 2 SD.				
		**Exclusion Criteria:** Patients diagnosed with dengue fever or other viral infections.	**Results:** Univariate analysis revealed that the significant risk factors for DSS were obesity, secondary infection type, platelet count < 20,000/uL, plasma leakage with hematocrit increase > 25% and inadequate fluid management from prior hospitalization. In multivariate analysis, obesity was not a risk factor for DSS, but plasma leakage with hematocrit increase > 25% was a risk factor.				
			**Exposure**	**DSS (%)**	**Non-DSS (%)**	**OR (95% CI)**	**P**
			Non-obese	93 (80.2%)	201 (88.9%)	-	-
			Obese > + 2 SD	23 (19.8%)	25 (11.1%)	1.88 (1.01, 3.51)	0.07
Lovera et al., 2016, Paraguay, N = 471 patients, Cohort [47].	To analyze the clinical and laboratory characteristics, risk factors and outcome of DSS in children.	**Inclusion Criteria:** Children admitted to Institute of Tropical Medicine with DF during '11-'13 outbreak of serotype 2.	**Methods:** This study uses the WHO 2009 dengue classification. There were 354 patients in the case group (patient with dengue shock syndrome) and 117 patients in the control group (patients with warning signs or severe dengue without shock). **Definition of Exposures:** Not mentioned.				
		**Exclusion Criteria**: Patients with hematologic disorders, congenital heart diseases, congenital or acquired immunodeficiency, cancer, and chronic lung or renal diseases.	**Results:** DS not associated with malnutrition, obesity or overweight.				
			**Exposure**	**Shock (%)**	**Non-shock (%)**	**OR (95% CI)**	**P**
			Obese	21 (5.9%)	5 (4%)	1.40 (0.50, 4.00)	0.49
			Obese and Overweight	97 (27%)	27 (23%)	1.20 (0.70, 2.00)	0.35
			Malnutrition	41 (11.6%)	16 (14%)	0.8 (0.40, 1.50)	0.54
			Severe Malnutrition	3 (0.8%)	4 (3.4%)	0.2 (0.00, 1.00)	0.06
Tatura et al., 2016, Indonesia, N = 58 patients, Cohort [48].	To assess IL-8 levels in DHF patients and determine the correlation between IL-8 concentration on admission and DSS outcomes.	**Inclusion Criteria:** Pediatric patients aged 1–14 years and diagnosed with DSS, according to the WHO (2011) criteria.	**Methods:** This study uses the WHO 2011 dengue classification. There were 27 patients in the case group (deterioration in dengue shock syndrome) and 31 patients in the control group (improvement in dengue shock syndrome). **Definition of Exposures:** Not mentioned.				
		**Exclusion Criteria:** Children with a bacterial infection and those who had received corticosteroid treatment or blood transfusion.	**Results:** Normal nutritional status in 19/31 children in the DSS improvement group and 20/27 children in the DSS deterioration group.				
			**Exposure**	**Improvement (%)**	**Deterioration (%)**		
			Obese	5 (16.1%)	2 (7.4%)		
			Overweight	3 (9.7%)	1 (3.7%)		
			Normal	19 (61.3%)	20 (74.1%		
			Undernutrition	4 (12.9%)	3 (11.1%)		
			Malnutrition	0 (0.0%)	1 (3.7%)		
Kalayanarooj et al., 2005, Thailand, N = 4,532 patients, Case-control [10].	To discover whether the nutritional status has any effect on the severity of the dengue illness.	**Inclusion Criteria:** Confirmed dengue patients at Queen Sirikit National Institute of Child Health with body weights on admission.	**Methods:** This study uses the WHO 1997 dengue classification, and the exposures were determined using the weight-for-age percentile. 1,123 patients in the DSS Group (patient with dengue hemorrhagic fever grade III and IV). 2,544 patients in the DHF group (patient with dengue hemorrhagic fever grade I and II). 865 patients in the DF group (patient with dengue fever), and 734 patients in the Control group (patient admitted to the dengue ward during the same period with other diagnoses excluding HIV/AIDS). **Definition of Exposures: a. Obese =** >110% ideal body weight-for-age standard for Thai children.				
		**Exclusion criteria:** Not mentioned	**Results:** About 23–24% of the patients were overweight/obese, and control patients with other diagnoses had fewer obese patients (12.5%). Compared to control, there is a significant association between obesity and dengue infection (p <0.05). Obese patients had more unusual presentations and complications compared to normal and malnourished patients, such as encephalopathy (1.3% vs 0.5% and 1.2%), associated infections (4.8% vs 2.7% and 3.1%), and fluid overload (6.5% vs 3.2% and 2.1%).				
		** **	**Exposure**	**DF (%)**	**DHF (%)**	**DSS (%)**	**Control (%)**
			Malnourished	96 (11.1%)	201 (7.9%)	122 (10.9%)	144 (19.6%)
			Normal	566 (65.4%)	1,720 (67.6%)	732 (65.2%)	498 (67.9%)
			Obese	203 (23.5%)	623 (24.5%)	269 (24.0%)	92 (12.5%)
Pichainarong et al., 2006, Thailand, N = 210 patients, Case-control [31].	To evaluate effects of childhood, caregivers, environmental, and etiological factors on the severity of DF/DHF in children of various body sizes.	**Inclusion Criteria:** 0–14 years old patients admitted to Queen Sirikit National Institute of Child Health, Bangkok between Oct '02 and Nov '03.	**Methods:** This study uses the WHO 1999a dengue classification, and the exposure was determined using the weight-for-age with standard deviation (SD). There were 105 patients in the case group (patient with dengue hemorrhagic fever grade III and IV) and 105 patients in the control group (patient with dengue hemorrhagic fever grade I and II). **Definition of Exposures: a. Obese =** Weight-for-age 1.5 SD above mean. * Additional details on the number of events among cases and control were obtained from the author by email.				
		**Exclusion Criteria:** Final diagnosis by the physician was DF or viral infection, or there was no data for weights and heights.	**Results:** The patients who had obesity were at increased risk for more severe DHF (OR = 2.77, 95% CI 1.19, 6.45) compared to those at a normal weight.				
			**Exposure**	**Cases (%)**	**Control (%)**	**OR (95% CI)**	**P**
			Normal	22 (30%)	31 (29.5%)	1	-
			Thin	60 (57.1%)	65 (61.9%)	0.77 (0.38, 1.55)—adjusted	0.45—adjusted
			Obese	23 (21.9%)	9 (8.6%)	3.00 (1.20, 7.48)—adjusted	< 0.01—adjusted
Junia et al., 2007, Indonesia, N = 600 patients, Case-control [40].	To determine the clinical risk factors for DSS.	**Inclusion Criteria:** DHF and DSS patients age less than 14 years, admitted to the hospital from Jan ‘04 to Dec ‘05.	**Methods:** This study uses the WHO 1997 dengue classification, and the exposures were determined using the weight-for-height (WH) percentage (%). There were 200 patients in the case group (patient with dengue hemorrhagic fever grade III and IV) and 400 patients in the control group (patient with dengue hemorrhagic fever grade I and II). **Definition of Exposures: a. Obese =** Weight/Height percentage more than 110%.				
		**Exclusion Criteria:** History of bronchial asthma, diabetes mellitus, sickle cell anemia, typhoid fever, sepsis, and measles.	**Results:** Factors associated with risk factors for DSS were aged 5–9 years, overweight, prolonged vomiting, persistent abdominal pain, and massive bleeding. All the independent variables were analyzed using logistic regression for p < 0.25 except for sex. In multivariate analysis, aged 5–9 years, overweight, and persistent abdominal pain were significant risk factors for DSS (p < 0.01).				
			**Exposure**	**Cases (%)**	**Control (%)**	**OR (95% CI)**	**P**
			Obesity	18 (9%)	33 (8,2%)	-	-
			Overweight	47 (23.5%)	55 (13.8%)	-	-
			Well-nourished	79 (39.5%)	201 (50.2%)	-	-
			Undernourished	56 (28%)	111 (27.8%)	-	-
			Univariate analysis (overweight/obese)	65 (32.5%)	88 (22.0%)	1.88 (1.22, 2.90)	0.01
Tantracheewathorn et al., 2007, Thailand, N = 165 patients, Case-control [41].	To determine the risk factors of dengue shock syndrome in children.	**Inclusion Criteria:** Children 15 years old or under who were compatible with definitions of DHF/DSS.	**Methods:** This study uses the WHO 1997 dengue classification, and the exposure was determined using the weight-for-age (WA) percentage (%). There were 55 patients in the case group (patient with dengue hemorrhagic fever grade III and IV) and 110 patients in the control group (patient with dengue hemorrhagic fever grade I and II). **Definition of Exposures: a. Obese =** Weight-for-age percentage more than 120%.				
		**Exclusion Criteria:** Patients with underlying hematologic diseases or other simultaneous infections.	**Results:** The age, sex, nutritional status, and duration of fever between both groups were not statistically different. Risk factors of DSS were bleeding, secondary dengue infection, and hemoconcentration of more than 22% from baseline hematocrit (adjusted OR (95%CI): 5.1 (1.50, 17.1), 21.8 (5.30, 90.8), 15.5 (4.40, 54.6), respectively).				
			**Exposure**	**Cases (%)**	**Control (%)**		
			Normal	24 (43.6%)	46 (41.8%)		
			Protein Energy Malnutrition	15 (27.3%)	37 (33.6%)		
			Obesity	16 (29.1%)	27 (24.5%)		
Putra et al., 2014, Indonesia, N = 94 patients, Case-control [46].	To assess for an association between serum transaminase levels and the presence of DSS in children.	**Inclusion Criteria:** All children with a diagnosis of suspected dengue infection according to the WHO 1997 criteria.	**Methods:** This study uses the WHO 1997 dengue classification. There were 47 patients in the case group (patient with dengue hemorrhagic fever grade III and IV) and 47 patients in the control group (patients with dengue hemorrhagic fever grade I and II). **Definition of Exposures:** Not mentioned.				
		**Exclusion Criteria:** Diagnosed with malignancies, immune disorders, hemato-oncology disorders, or history of hepatitis.	**Results:** Elevated AST and ALT levels associated with an increased risk of DSS in children with dengue infection.				
			**Exposure**	**Cases (%)**	**Control (%)**		
			Underweight	12 (26%)	9 (19%)		
			Normal Weight	24 (51%)	32 (68%)		
			Overweight	11 (23%)	6 (13%)		
Widagdo, 2008, Indonesia, N = 45 patients, Cross-sectional [43].	To investigate the relationship between blood zinc levels and the severity of DHF.	**Inclusion Criteria:** Fulfilled the clinical criteria for DHF and had a measurement of a serum zinc level.	**Methods:** This study uses the WHO 1999 dengue classification, and the exposure was determined using BMI-for-age. 29 patients in the DHF I group (patients with dengue hemorrhagic fever grade I). 12 patients in the DHF II group (patients with dengue hemorrhagic fever grade II). 2 patients in the DHF III group (patients with dengue hemorrhagic fever grade III), and 2 patients in the DHF IV group (patients with dengue hemorrhagic fever grade IV). **Definition of Exposures:** Not mentioned (BMI).				
		**Exclusion Criteria:** Failure to meet criteria.	**Results:** The average body weight was 20 ± 7.4 kg. Average height of 115 ± 19 cm and an average body mass index of 15.1 ± 3.2 kg/m2, slightly lower than the average for age. Average nutritional status of being under-nutrition (mild, moderate, or severe) in 20 children (44%), normal nutrition in 21 children (47%), and over-nutrition in 4 children (9%).				
			**Exposure**	**DHF I (%)**	**DHF II (%)**	**DHF III (%)**	**DHF IV (%)**
			Over-nutrition	2 (6.9%)	1 (8.3%)	0 (0%)	1 (50%)
			Normal	13 (44.8%)	5 (41.6%)	*2 (100%)*	1 (50%)
			Under-nutrition	14 (41.4%)	6 (50%)	0 (0%)	0 (0%)

### Study objectives of included studies

There was a broad range of objectives amongst the included studies. Three studies analyzed the association between nutritional status with dengue severity [10, 42, 44]. Five studies assessed the risk factors for dengue severity and dengue shock syndrome among children infected with dengue [31, 38, 40, 41, 47]. Remaining studies evaluated childhood obesity as a prognostic factor for dengue shock syndrome [45]; described the clinical disease patterns among children hospitalized for dengue [39]; determined the transfusion requirement among dengue infected patients [36]; analyzed the factor involved in thrombocytopenia and platelet aggregation among dengue hemorrhagic fever patients [37]; examined the association between blood zinc levels and its connection to the severity of dengue hemorrhagic fever clinically [43]; assessed the association between serum transaminase levels and the presence of dengue shock syndrome among children [46]; and determined the levels and correlation between interleukin-8 concentration on admission and dengue shock syndrome outcomes in children [48].

### Sample size and inclusion criteria used in the included studies

The fifteen studies included a total of 7,133 dengue patients with age ranged between 0 and 18 years old. All studies had a clear description of the inclusion criteria. Eleven studies included laboratory-confirmed dengue cases, and the other four [36, 43, 46, 48] studies did not report on the method used for confirmation of dengue cases. The diagnosis was confirmed using either serological test (hemagglutination inhibition or ELISA), viral isolation, or polymerase chain reaction (PCR) test.

### Exposures, tools of measurement, and measures of association

From all fifteen studies, ten studies used obesity [10, 31, 37, 39–42, 45, 47, 48], four studies used overweight [36, 38, 44, 46], and one study used over-nutrition [43] as the exposure. The measurement of obesity, overweight, and over-nutrition differ amongst studies. Six studies utilized weight-for-age [10, 31, 36, 38, 41, 42], three studies used BMI-for-age [39, 43–45], one study used weight-for-height [40], while five other studies did not mention the method used to measure the exposure [37, 46–48].

One in fifteen studies evaluated the possibility of obesity as a prognostic factor for dengue shock syndrome [45]. Two studies used nutritional status as their primary exposure for dengue severity [10, 44]. Seven studies analyzed the risk factors for dengue severity which include age, sex, nutritional status, as well as other clinical and laboratory risk factors [31, 38–42, 47]. In remaining five studies, the author presented obesity, overweight, or over-nutrition as ancillary results [36, 37, 43, 46, 48].

### Primary outcome and dengue classification

All five case-control studies defined dengue shock syndrome as the primary outcome [10, 31, 40, 41, 46]. For cohort study, five studies considered dengue shock syndrome as their primary outcome [36, 38, 45, 47, 48]. The remaining four studies used dengue hemorrhagic fever [42, 44] and severe dengue infection (dengue hemorrhagic grade III and IV) [37, 39] as their primary outcomes. While in the cross-sectional study [43], the author described the proportion of over-nutrition among all grade of dengue fever (grade I—IV).

All fifteen studies adopted various World Health Organization classifications of dengue. One study used the WHO 1986 criteria [36]. Nine studies used the WHO 1997 criteria [10, 37, 38, 40–42, 44–46]. Two studies used the WHO 1999 criteria [39, 43]. One study used the WHO 1999a criteria [31]. One study used the WHO 2009 criteria [47], and one study used the WHO 2011 criteria [48].

### Quality of studies

According to the assessment of study quality using the Newcastle-Ottawa Quality Assessment Scale, we classified nine studies as good quality studies [10, 31, 37–39, 42–44, 48] and six studies as moderate quality studies [36, 40, 41, 45–47]. Table 3 summarized the total score acquired for each quality domain. Based on the selection domain, six studies [36, 40, 41, 45–47] scored two out of four points, six studies [38, 44–48] scored three out of four points, and three studies [37, 39, 42] scored four over four quality score points. In the comparability domain, eleven studies [10, 31, 36, 38, 40–43, 45–47] scored one over two points, and four studies [37, 39, 44, 48] scored a full point of two over two. In the exposure domain, all fifteen studies scored three out of four quality score points. By study design, eight [37–39, 42, 44, 45, 47, 48] out of nine cohort studies were classified as good quality studies and one [36] as a moderate quality study. Furthermore, one [46] case-control study was classified as good quality study while four case-control [10, 31, 40, 41] studies were classified as moderate quality. The only cross-sectional study [43] in this review was classified as a moderate quality study.

**Table 3:** Quality assessment score points.

First Author	Study Year	Quality Score Points			
		Selection	Comparability	Exposure	Total
Chuansumrit et al.	2000	2	1	3	6
Basuki	2003	4	2	3	9
Kan et al.	2004	3	1	3	7
Kalayanarooj et al.	2005	2	1	3	6
Pichainarong et al.	2006	2	1	3	6
Malavige et al.	2006	4	2	3	9
Junia et al.	2007	2	1	3	6
Tantracheewathorn et al.	2007	2	1	3	6
Bongsebandhu et al.	2008	4	1	3	8
Widagdo	2008	2	1	3	6
Maron et al.	2010	3	2	3	8
Widiyati et al.	2013	3	1	3	7
Putra et al.	2014	3	1	3	7
Lovera et al.	2016	3	1	3	7
Tatura et al.	2016	3	2	3	8

### Summary of the results of the studies

#### Cross-sectional study

The only cross-sectional study in this review investigated the association between blood zinc levels and dengue severity. Primarily, the author found no association between zinc levels and clinical severity of DHF in children. However, based on the available descriptive analysis results, a higher average of body mass index (BMI) among those with DHF grade IV was found (BMI 18.8 ± 7.2).

#### Case-control studies

Among five case-control studies, two studies suggested an association between obesity and dengue severity [31, 40]. Both studies found higher odds of dengue severity among those who were obese. Moreover, Junia et al. also found that overweight was among the risk factors for dengue shock syndrome together with age five to nine years old, prolonged vomiting, persistent abdominal pain, and massive bleeding [40].

In contrast, three remaining case-control studies found no significant association between obesity and dengue severity [10, 41, 46]. However, Kalayanarooj et al. found a higher percentage of the overweight and obese patient among their study participants. The author also found that obese patients have a more unusual presentation and complications compared to those in the normal and malnourish group. Furthermore, complication such as encephalopathy (1.3%, 0.5% and 1.2%), associated infections (4.8%, 2.7% and 3.1%), and fluid overloaded (6.5%, 3.2%, and 2.1%) were also found among obese patients.

#### Cohort studies

Among all included cohort studies, five studies analyzed the potential risk factors for dengue severity [38, 42, 44, 45, 47] Three other studies did not measure the association between obesity and dengue severity as their primary objective [36, 37, 48].

Six studies found no significant association between obesity and dengue hemorrhagic fever as well as dengue shock syndrome [38, 39, 44, 45, 47, 48]. Widiyati et al. who evaluated the role of childhood obesity as a prognostic factor for dengue shock syndrome, concluded that obesity was not a significant risk factor for dengue shock syndrome (OR = 1.03; 95% CI:0.32,3.31; p = 0.07) [45]. Similarly, Malavige et al. concluded that patients with BMI-for-age above 90^th^ centile did not seem to modify the risk of severe dengue infection [39]. Maron et al. also found no differences in the BMI-for-age Z-scores or weight-for-age between dengue fever, dengue hemorrhagic fever, and healthy control group [44]. In contrast, three cohort studies found a higher percentage of obese patients among the severe group when compared to non-severe group [36, 37, 42]. Chuansumrit et al. concluded that the occurrence of dengue hemorrhagic fever grade III or IV was more prominent in patients whose body weight exceeds the 50^th^ percentile for age [36]. Similarly, Bongsebandhu et al. and Basuki et al. found a higher percentage of obese among dengue hemorrhagic fevers patients [37, 42].

### Meta-analysis

**Fig 2:**
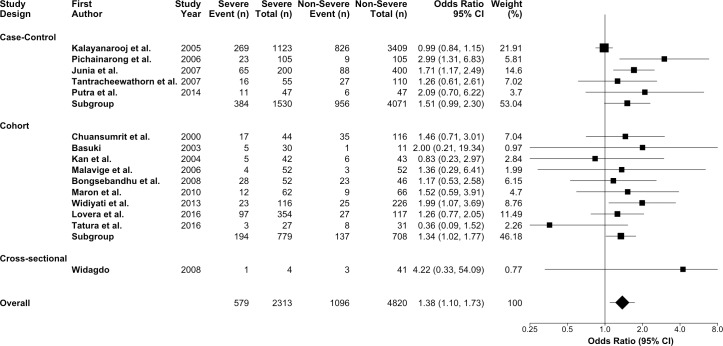
Forest plot for the association between obesity and dengue severity.

**Fig 3:**
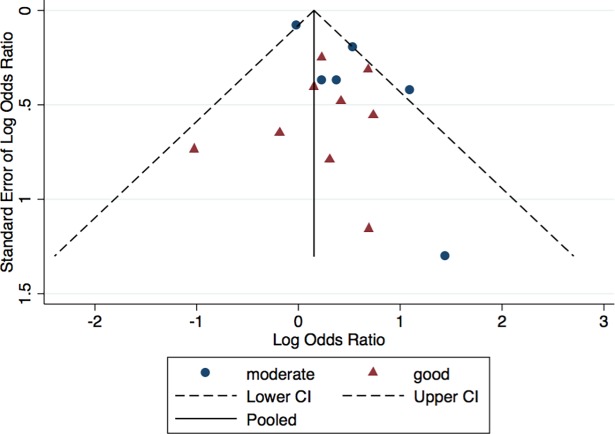
Funnel plot for the association between obesity and dengue severity.

We first pooled the odds ratio for all fifteen studies [10, 31, 36–48] evaluating the association between obesity and dengue severity. We found that dengue patients who were obese have 38 percent higher odds of developing a severe presentation of dengue infection compared to non-obese dengue patients (OR = 1.38; 95% CI:1.10,1.73; p = 0.01; I^2^ = 36.7%) (Fig 2). We found no significant heterogeneity between studies (p = 0.08). There was no statistical evidence of publication bias based on the funnel plot (Fig 3) and Egger’s Test (p = 0.06). In sensitivity analyses using meta-regression, there was no statistical evidence of heterogeneity in obesity classification (p = 0.72), study quality (p = 0.84), nor study design (p = 0.67).

**Fig 4:**
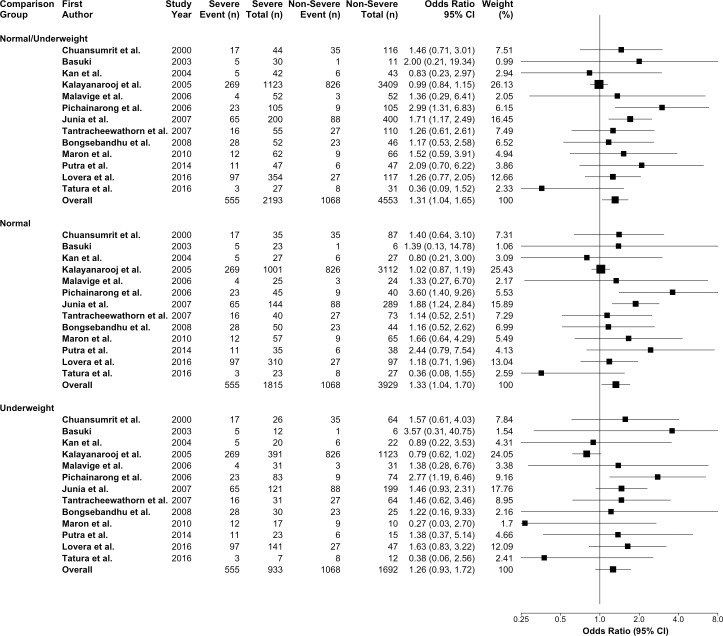
The secondary analysis on the association between obesity and dengue severity comparing with non-obese, normal weight, and underweight groups.

As a secondary analysis, we compared obese group in thirteen studies [10, 31, 36–42, 44, 46–48] with different comparison groups that include: (i) normal weight and underweight; (ii) normal weight; and (iii) underweight. From the pooled analyses, we found that the odds of developing severe dengue infection among the obese group were lower and not statistically significant when compared with the underweight group (OR = 1.26; 95% CI:0.93,1.72; p = 0.14). A significant higher odds ratio was found when we compared obese to normal/underweight group (OR = 1.31; 95% CI:1.04,1.65; p = 0.02) and normal weight group (OR = 1.33; 95% CI:1.04,1.70; p = 0.03) (Fig 4).

## Discussion

Our systematic review found a significant association between obesity and dengue severity among children. We found that there were 38 percent higher odds of developing severe dengue infection among obese dengue patients compared to non-obese patients. The results of this review support our hypothesis that obese patients have more severe dengue infection compared to non-obese patients. These findings may be useful in the identification of high-risk dengue patients. Based on our literature review, obese patients were found to be at high risk of developing complication and death due to their stronger immune response compared to malnourished patients [10]. Hypothetically, obesity may affect the severity of dengue through inflammation pathways. The increased deposition of white adipose tissue (WAT) in obese individuals leads to an increased production of Interleukin-six (IL-6), Interleukin-eight (IL-8), and Tumor Necrosis Factor-Alpha (TNF-α) [25–27]. IL-6, IL-8, and TNF-a were essential mediators of the inflammation pathway that increases capillary permeability.

An increased capillary permeability in obese dengue patients may progressively underlie the process of severe plasma leakage. Thus, with increasing prevalence of obesity in regions with high-risk of dengue infection and the hypothesized link between obesity and dengue severity, an early detection of obese dengue patients was required for closer monitoring and early treatment.

Our systematic review found that the included studies defined and measured obesity differently using a different index of measurements such as BMI-for-age, weight-for-age, and weight-for-height. We also found that there were differences in study quality and study design. However, based on our statistical analysis, no heterogeneity found between obesity classification, study quality, and study design. Thus, we concluded that the differences in obesity classification, study quality, and study design do not modify the association between obesity and severity of dengue infection.

There were also differences in types of dengue classification used between studies. Since 2009, multiple revisions on the dengue classification have been done by the World Health Organization (WHO) to improve dengue patient management. Currently, the latest classification of dengue categorizes dengue patient into dengue fever, dengue with warning signs, dengue without warning signs and severe dengue. Most studies in this review were using the WHO 1997 dengue classification. Among all, only two studies [47, 48] used the latest WHO classification (2009, 2011) to classify their dengue cases. We did not reclassify cases using the WHO 2009 classification due to the unavailability of the raw data and also to the fact that the WHO 2009 classification classified dengue severity differently based on the presence of warning signs and severe dengue. Thus, with currently available information, it was difficult for us to reclassify all patients as it may lead to selection bias towards the more severe group.

Apart from that, all studies included in this review focused only on subjects age between 0 and 18 years old. The unavailability of adult patients limits the applicability of our findings to the general population. With regards to study settings, most of the included studies focus only on hospitalized patients. The hospital settings will encounter more severe dengue cases than primary health care settings. By restricting the study subject to hospitalized patients might introduce bias. It also limits the analysis to a more limited severe dengue group and left out the non-hospitalized group which might be less severe. Therefore, studies related to risk factors or prognostic factors should also include primary healthcare facilities.

Based on our literature review, there were higher risks of developing DHF and DSS among individuals with co-morbidities such as diabetes and hypertension[49]. We believed the association between these co-morbidities might be an effect modifier that increases the risk of severe dengue infection but limited only to adult patients. The pathophysiology underlying the effects was not clearly understood as there were not many earlier studies were available. We also found that significantly higher rates of general and central obesity were reported among men and women with higher socioeconomic status in South Asian nations [50, 51]. In most of the developing countries of South Asia region, the magnitude of household out-of-pocket expenditures on health was at times as high as 80 percent of the total amount spent on health care per annum [52].

We believed that there was a better health-seeking behavior among obese individuals compared to a non-obese individual due to their accessibility for better health care options. Thus, more obese patients were recorded from hospital compared to non-obese patients. We could not ascertain the association between the presence of co-morbidities among obese patients as well as effects of the socioeconomic status of obese patients and severity of dengue infection due to scarce information available.

This review has its limitations. We acknowledged the misclassification of obesity due to our inclusion of overweight and over-nutrition. However, we expect the bias to be towards the null as both overweight and over-nutrition refer to similar measures of body composition that is above normal weight. Furthermore, we accepted that the exclusion of fifteen patients from the study by Chuansumrit et al. was due to unavailability of body measurement data [36]. Another seventy-four [44] and seven-hundred-thirty-four controls [10] were also not included because our study does not include the non-dengue patients. Moreover, we also acknowledged that no study with adult participants was included in this review. The unavailability of studies with adult participants might be because previously the distributions of dengue infection were more prevalent among children and less severe among adults.

Also, all fifteen included studies were heterogeneous regarding classification, exposure, and outcomes, thus making it more challenging to compare and infer the results. However, these problems were adequately addressed by including all studies that associated with the research question, and not excluding it for lack of quality.

## Conclusion

In conclusion, our systematic review and meta-analysis identified a significant association between obesity and dengue severity. The results presented highlight the importance of obesity in dengue infection. It also improves our understanding of the effect of obesity in influencing the severity of dengue infection among children. Further large-scale prospective cohort studies on the effects of obesity on dengue severity among adults and children especially in regions with high prevalence of dengue could provide additional information as well as better understanding on the relevance of obesity in dengue infection.

## Supporting information

S1 File PRISMA 2009 checklist. (PDF)

S2 File PRISMA 2009 Flow Diagram. (PDF)

S1 Table Cut-off score points for study quality assessment. (PDF)

S2 Table Search strategy results. (PDF)

S3 Table Calculated odds ratio of individual study. (PDF)
