# Supplementary material for: The association between obesity and dengue severity among pediatric patients: A systematic review and meta-analysis
Source: PLoS Negl Trop Dis. 2018 Feb 7;12(2):e0006263. doi: 10.1371/journal.pntd.0006263 (PMC5819989; doi:10.1371/journal.pntd.0006263)
Supplement: S2 File — (PDF) [file pntd.0006263.s002.pdf]

PRISMA 2009 Flow Diagram

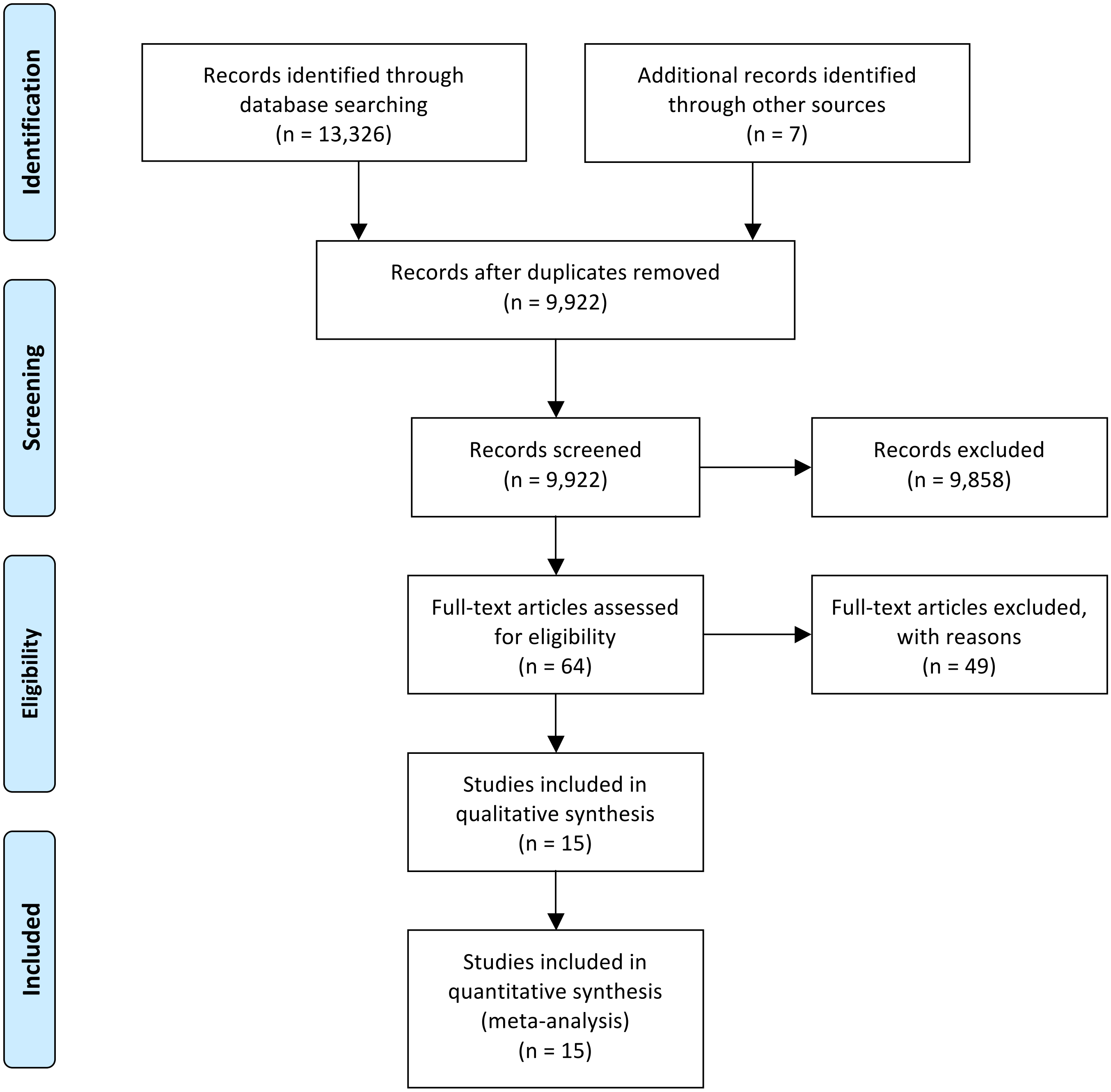

From: Moher D, Liberati A, Tetzlaff J, Altman DG, The PRISMA Group (2009). *P*referred *R*eporting *I*tems for *S*ystematic Reviews and *M*eta-*A*nalyses: The PRISMA Statement. PLoS Med 6(7): e1000097. doi:10.1371/journal.pmed1000097

For more information, visit www.prisma-statement.org.
