# Supplementary material for: The association between obesity and dengue severity among pediatric patients: A systematic review and meta-analysis
Source: PLoS Negl Trop Dis. 2018 Feb 7;12(2):e0006263. doi: 10.1371/journal.pntd.0006263 (PMC5819989; doi:10.1371/journal.pntd.0006263)
Supplement: S1 Table — (PDF) [file pntd.0006263.s003.pdf]

S1 Table: Cut-off Score Points for Study Quality Assesment

Group	Cut off Score		
	**Good**	**Moderate**	**Poor**
**Selection**	≥ 3	2	1
**Comparability**	2	1	0
**Exposure**	≥ 3	2	1
**Total Points**	≥ 7	≥ 5	≥ 4

Above cut-off score was adapted from McPheeters et al. 2012 report on Quality Improvement Interventions To Address Health Disparities. (Appendix G page 103-104 in http://www.ncbi.nlm.nih.gov/pubmedhealth/PMH0049229/)
