# Supplementary material for: The association between obesity and dengue severity among pediatric patients: A systematic review and meta-analysis
Source: PLoS Negl Trop Dis. 2018 Feb 7;12(2):e0006263. doi: 10.1371/journal.pntd.0006263 (PMC5819989; doi:10.1371/journal.pntd.0006263)
Supplement: S2 Table — (PDF) [file pntd.0006263.s004.pdf]

#	Ovid (Medline)	No. of Articles	Embase	No. of Articles
**1**	dengue.sh.	9,381	dengue	21,601
**2**	dengue.ti.	12,368	'dengue'/exp	14,943
**3**	dengue.ab.	14,527	adipos*	137,289
**4**	adipos*.sh.	85,986	'adiposity'/exp	379,989
**5**	adipos*.ti.	31,842	obes*	436,404
**6**	adipos*.ab.	81,047	'obesity'/exp	379,989
**7**	obes*.sh.	174,412	'body mass index'	180,734
**8**	obes*.ti.	103,571	'body mass index'/exp	256,911
**9**	obes*.ab.	219,105	bmi	203,359
**10**	body mass index/.sh.	107,153	'waist circumference*'	39,505
**11**	body mass index/.ti.	12,313	'waist circumference'/exp	34,879
**12**	body mass index/.ab.	144,019	nutriti*	714,868
**13**	bmi.sh.	0	'nutrition'/exp	1,801,875
**14**	bmi.ti.	4,131	malnutriti*	64,822
**15**	bmi.ab.	111,517	'malnutrition'/exp	137,445
**16**	waist circumference*/.sh.	7,939	flavi*	63,963
**17**	waist circumference*/.ti.	1,619	'flavivirus'/exp	24,224
**18**	waist circumference*/.ab.	21,571	#1 OR #2	21,601
**19**	nutriti*.sh.	134,472	#3 - #17/OR	2,700,072
**20**	nutriti*.ti.	93,489	#18 AND #19	11,564
**21**	nutriti*.ab.	191,555	#20 AND [humans]/lim	7,160
**22**	malnutriti*.sh.	10,891		
**23**	malnutriti*.ti.	10,692		
**24**	malnutriti*.ab.	28,352		
**25**	flavi*.sh.	11,790		
**26**	flavi*.ti.	5,896		
**27**	flavi*.ab.	17,546		
**28**	#1 or #2 or #3	17,727		
**29**	#4 - #27/or	807,166		
**30**	#28 and #29	2,188		
**31**	limit 30 to humans	1,275		

#	Cochrane	No. of Articles	Scopus	No. of Articles
**1**	dengue:ti,ab,kw	327	(TITLE-ABS-KEY(dengue))	23,272
**2**	adipos*:ti,ab,kw	4,661	(TITLE-ABS-KEY(adipos*))	151,350
**3**	obes*:ti,ab,kw	26,156	(TITLE-ABS-KEY(obes*))	389,750
**4**	'body mass index':ti,ab,kw	24,318	(TITLE-ABS-KEY('body mass index'))	217,123
**5**	bmi:ti,ab,kw	18,064	(TITLE-ABS-KEY(bmi))	124,707
**6**	'waist circumference*':ti,ab,kw	4,541	(TITLE-ABS-KEY('waist circumference'))	34,296
**7**	nutriti*:ti,ab,kw	43,825	(TITLE-ABS-KEY(nutriti*))	584,005
**8**	malnutriti*:ti,ab,kw	2,654	(TITLE-ABS-KEY(malnutriti*))	69,516
**9**	flavi*:ti,ab,kw	524	(TITLE-ABS-KEY(flavi*))	60,790
**10**	#2 - #9/or	83,721	#2 - #9/OR	1,237,329
**11**	#1 and #10	48	#1 AND #10	3,688
**12**			#11 AND (LIMIT-TO (EXACTKEYWORD , "Human"))	2,077
**13**				
**14**				
**15**				
**16**				
**17**				
**18**				
**19**				
**20**				
**21**				
**22**				
**23**				
**24**				
**25**				
**26**				
**27**				
**28**				
**29**				
**30**				
**31**				

#	Web of Science	No. of Articles
**1**	TS=dengue	19,064
**2**	TI=dengue	11,794
**3**	TS=adipos*	118,218
**4**	TI=adipos*	36,226
**5**	TS=obes*	305,731
**6**	TI=obes*	120,232
**7**	TS='body mass index'	163,965
**8**	TI='body mass index'	16,165
**9**	TS=bmi	102,087
**10**	TI=bmi	7,636
**11**	TS='waist circumference'	22,803
**12**	TI='waist circumference'	2,212
**13**	TS=nutriti*	311,477
**14**	TI=nutriti*	100,074
**15**	TS=malnutriti*	30,961
**16**	TI=malnutriti*	8,288
**17**	TS=flavi*	26,775
**18**	TI=flavi*	7,878
**19**	#1 OR #2	19,064
**20**	#3 - #18/OR	793,751
**21**	#19 AND #20	2,989
**22**	#19 AND #20 NOT TS=animal	2,766
**23**		
**24**		
**25**		
**26**		
**27**		
**28**		
**29**		
**30**		
**31**		
