# Supplementary material for: The association between obesity and dengue severity among pediatric patients: A systematic review and meta-analysis
Source: PLoS Negl Trop Dis. 2018 Feb 7;12(2):e0006263. doi: 10.1371/journal.pntd.0006263 (PMC5819989; doi:10.1371/journal.pntd.0006263)
Supplement: S3 Table — (PDF) [file pntd.0006263.s005.pdf]

S3 Table: Calculated Odds Ratio (OR) of individual studies.

Study ID	Year	Severe		Non-Severe		Odds Ratio
		Obese/Overweight	Normal/Underweight	Obese/Overweight	Normal/Underweight	
**Chuansumrit et al.**	**2000**	17	27	35	81	1.46
**Basuki**	**2003**	5	25	1	10	2.00
**Kan et al.**	**2004**	5	37	6	37	0.83
**Kalayanarooj et al.**	**2005**	269	854	826	2583	0.99
**Pichainarong et al.**	**2006**	23	82	9	96	2.99
**Malavige et al.**	**2006**	4	48	3	49	1.36
**Tantracheewathorn et al.**	**2007**	16	39	27	83	1.26
**Junia et al.**	**2007**	65	135	88	312	1.71
**Widagdo**	**2008**	1	3	3	38	4.22
**Bongsebandhu et al.**	**2008**	28	24	23	23	1.17
**Maron et al.**	**2010**	12	50	9	57	1.52
**Widiyati et al.**	**2013**	23	93	25	201	1.99
**Putra et al.**	**2014**	11	36	6	41	2.09
**Lovera et al.**	**2016**	97	257	27	90	1.26
**Tatura et al.**	**2016**	3	24	8	23	0.36
